# *Lactobacillus*-Depleted Vaginal Microbiota in Pregnant Women Living With HIV-1 Infection Are Associated With Increased Local Inflammation and Preterm Birth

**DOI:** 10.3389/fcimb.2020.596917

**Published:** 2021-02-11

**Authors:** Charlotte-Eve S. Short, Richard G. Brown, Rachael Quinlan, Yun S. Lee, Ann Smith, Julian R. Marchesi, Robin Shattock, Phillip R. Bennett, Graham P. Taylor, David A. MacIntyre

**Affiliations:** ^1^ Department of Infectious Disease, Section of Virology, Imperial College London, London, United Kingdom; ^2^ St Mary’s Hospital, Imperial College Healthcare NHS Trust, London, United Kingdom; ^3^ Department of Metabolism, Digestion, and Reproduction, March of Dimes Prematurity Research Centre, Division of the Institute of Reproductive and Developmental Biology, Faculty of Medicine, Imperial College London, London, United Kingdom; ^4^ Faculty of Health and Applied Sciences, University West of England, Bristol, United Kingdom; ^5^ School of Biosciences, Cardiff University, Cardiff, United Kingdom; ^6^ Department of Metabolism, Digestion, and Reproduction, Division of Digestive Disease, Faculty of Medicine, Imperial College London, London, United Kingdom; ^7^ Department of Infectious Disease, Section of Mucosal Infection and Immunity, Imperial College London, London, United Kingdom

**Keywords:** HIV, preterm, microbiome, *Lactobacillus* sp. *Gardnerella* spp., bacterial diversity, inflammation, cytokines

## Abstract

**Background:**

Pregnant women living with HIV-1 infection (PWLWH) have an elevated risk of preterm birth (PTB) of unknown aetiology, which remains after successful suppression of HIV. Women at high risk for HIV have a common bacterial profile which has been associated with poor birth outcomes. We set out to explore factors associated with gestational age at delivery of PWLWH in a UK population.

**Methods:**

Prospective study of PWLWH (n = 53) in whom the vaginal microbiota and cervicovaginal cytokine *milieu* were assessed using metataxonomics and multiplexed immunoassays, respectively. Cross-sectional characterisation of vaginal microbiota in PWLWH were compared with 22 HIV uninfected pregnant women (HUPW) at a similar second trimester timepoint. Within PWLWH the relationships between bacterial composition, inflammatory response, and gestational age at delivery were explored.

**Findings:**

There was a high rate of PTB among PWLWH (12%). In the second trimester the vaginal microbiota was more diverse in PWLWH than in HUPW (Inverse Simpson Index, p = 0.0004 and Species Observed, p = 0.009). PWLWH had a lower prevalence of *L. crispatus* dominant vaginal microbiota group (VMB I, 15 vs 54%) than HUPW and higher prevalence of *L. iners* dominant (VMB III, 36 vs 9% and VMB IIIB, 15 vs 5%) and mixed anaerobes (VMB IV, 21 vs 0%). Across the second and third trimesters in PWLWH, VMB III/IIIB and IV were associated with PTB and with increased local inflammation [cervicovaginal fluid (CVF) cytokine concentrations in upper quartile]. High bacterial diversity and anaerobic bacterial abundance were also associated with CVF pro-inflammatory cytokines, most notably IL-1β.

**Interpretation:**

There is an association between local inflammation, vaginal dysbiosis and PTB in PWLWH. Understanding the potential of antiretroviral therapies to influence this cascade will be important to improve birth outcomes in this population.

## Introduction

Preterm birth (<37 weeks completed gestation; PTB) affects in excess of 15 million women per year (Blencowe et al., 2012) and accounts for 35% of neonatal death worldwide (Liu et al., 2016). PWLWH are at greater risk of PTB than the general population (Uthman et al., 2017) with rates up to 40% reported in some cohorts (Grosch-Woerner et al., 2008; Short and Taylor, 2014). A high prevalence of recognised PTB risk factors such as Black race, low BMI, anaemia, and a past history of PTB are likely to contribute to this phenomenon. Additional factors associated with HIV-infection such as immune suppression and increased susceptibility to co-infections by pathogens such as cytomegalovirus, malaria, and human papillomavirus (HPV; +/- cervical interventions) may also have a role (Short and Taylor, 2014). Despite the advent of effective antiretroviral therapy (ART, usually three drugs combined) which enables immune reconstitution and reduces the rate of mother-to-child transmission of HIV (MTCT) to less than 1%, the rates of PTB have not declined (Thorne et al., 2004).

An association between ART and PTB has been repeatedly reported (Short and Taylor, 2014). The class of drugs and the timing of treatment in relation to conception appear to affect the risk, however, results from observational studies have been inconsistent (Short and Taylor, 2014; Uthman et al., 2017). Ritonavir-boosted lopinavir, a member of the protease inhibitor class, has been associated with a two-fold increase in PTB (Powis et al., 2011; Fowler et al., 2016). In spite this protease inhibitors remain a preferred option in many antenatal guidelines, including the UK, due to a greater experience in pregnancy with these agents compared to other newer drugs and their strong barrier to resistance (Gilleece et al., 2019). Conversely, zidovudine monotherapy, no longer used routinely, has been repeatedly associated with low rates of PTB (Short and Taylor, 2014). UNAIDS estimated that in 2017 there were 1 million pregnant women living with HIV in east and southern Africa of which 93% received antiretroviral therapy (UNAIDS, 2018). Lifelong ART is now recommended for all for its long-term health benefits, thus increasing the length of exposure, during pregnancy to all ART, including nucleoside analogues with antimicrobial properties, and the potential for adverse effects.

There is now substantial evidence that vaginal microbiota composition is an independent risk factor for PTB. Bacterial vaginosis (BV), microbiologically characterised by reduced levels of *Lactobacillus* species and overgrowth of anaerobic bacteria, has long been recognised to increase PTB risk (Hay et al., 1994). Recent studies using molecular-based metataxonomics profiling during pregnancy also show that vaginal microbiota dominated by *Lactobacillus* species, particularly *L. crispatus*, are stable and associate with protection against PTB, whereas increased prevalence of high-diversity communities depleted in *Lactobacillus* spp., and enriched for potential pathobionts such as *Gardnerella vaginalis, Sneathia* spp.*, Prevotella* spp., and members of the Mollicutes associate with increased risk (Kindinger et al., 2016; Brown et al., 2018; Brown et al., 2019; Elovitz et al., 2019; Fettweis et al., 2019). The latter “sub-optimal” community compositions are also prevalent in women with, or at risk of, HIV infection (Spear et al., 2008; Hummelen et al., 2010; Pepin et al., 2011; Borgdorff et al., 2014; Reimers et al., 2016) and represent a risk factor for sexual transmission of HIV and other infections (e.g., HPV and Herpes Simplex Virus) (Borgdorff et al., 2014; Reimers et al., 2016). They also associate with enhanced HIV shedding in cervicovaginal fluid (CVF) (Borgdorff et al., 2014) and may substantially reduce efficacy of topical pre-exposure prophylaxis preparations such as 1% tenofovir gel (Klatt et al., 2017).

The vaginal microbiota of PWLWH have been under investigated. In this study, we set out to characterise the vaginal microbiota of a UK cohort of PWLWH sampled longitudinally throughout gestation compared to HIV uninfected pregnant women (HUPW) using metataxonomic profiling. We also aimed to determine if differences in vaginal microbiota composition of PWLWH influence local inflammation and subsequent risk of PTB.

## Materials and Methods

### Study Design and Setting

This was a prospective and observational study of 53 PWLWH and 22 HUPW. Following written informed consent, women were recruited at 8–14 weeks gestation in HIV specialist and general antenatal clinics of ten London hospitals, UK between January 2013 and August 2017 (Barnet, Chelsea and Westminster, Homerton, Lewisham, North Middlesex, Northwick Park, Queen Charlotte, Queen Elizabeth, St Mary’s and St Thomas’ Hospital). The study was approved by the NHS Health Research Authority National Research Ethics Service (NRES) Committee approval (REC 13/LO/0107 (PWLWH) & REC 14/LO/0328 (HUPW).

### Participants and Sample Collection

Women were eligible if 18 years of age or older and had known HIV status with a singleton pregnancy (confirmed on ultrasound). Exclusion criteria were: CD4 cell count <350 cells/mm^3^ if a PWLWH; or co-morbidities requiring immune modulating treatment (to limit bias introduced by immunosuppression); current injecting drug use and fertilization *in vitro*. Clinical data on medical and obstetric risk factors for PTB, intrapartum management and birth outcome were recorded for all women. The practice of vaginal douching, recent sexual intercouse and antibiotic use were also recorded. CD4 cell count, plasma HIV RNA concentration and ART regimen were additionally documented for PWLWH. Screening for syphilis, gonorrhea and chlaymdial infection was routinely offered as per national guidelines (Gilleece et al., 2019).

Clinician or self-sampling of the high lateral vaginal wall was undertaken using a BBL ™ CultureSwab™ MaxV Liquid amies swab. For PWLWH sampling of vagina was undertaken at three time points: 16.0–21.9 (t1), 22.0–26.9 (t2), and 27–31.9 weeks (t3) and for HUPW vaginal sampling occurred at one second trimester time point. An additional sample of CVF was obtained from PWLWH at all time points using a soft cup (Instead™). All samples were snap frozen and stored at −80°C within 2 h of collection until further processing.

### DNA Extraction and 16S rRNA Gene Sequencing (Metataxonomics)

Bacterial DNA was extracted using a combination of enzymatic digestion and mechanical disruption of cell membranes and the QIAamp DNA Mini kit (Qiagen, Manchester, UK), as previously described (MacIntyre et al., 2015). The V1-V2 hypervariable regions of the 16s rRNA gene were amplified with a fusion primer set that includes four different 28F primers chosen to improve detection of *Bifidobacteriales* and a 388R primer (Frank et al., 2008). The 28F-YM forward primer (5′-GAGTTTGATCNTGGCTCAG-3′) was mixed in a ratio of 4:1:1:1 with 28F *Borrellia* (5′-GAGTTTGATCCTGGCTTAG-3′), 28F *Chloroflex* (5′-GAATTTGATCTTGGTTCAG-3′), and 28F *Bifido* (5′-GGGTTCGATTCTGGCTCAG-3′) (RTL Genomics Amplicon Diversity Assay List). The forward primers included an Illumina i5 adapter (5′-AATGATACGGCGACCACCGAGATCTACAC-3′), an 8-base-pair (bp) bar code and primer pad (forward, 5′-TATGGTAATT-3′). The 388R reverse primer (5′-TGCTGCCTCCCGTAGGAGT-3′) was constructed with an Illumina i7 adapter (5′-CAAGCAGAAGACGGCATACGAGAT-3′), an 8-bp bar code, a primer pad (reverse, 5′-AGTCAGTCAG-3′). The pair end multiplex sequencing was performed on an Illumina MiSeq platform (Illumina Inc.) at Research and Testing Laboratory (Lubbock, TX, USA).

### Sequence Analysis:

The MiSeq SOP pipeline and software package Mothur were used to analyse RNA sequence data. Highly similar amplicons were clustered into operational taxonomic units (OTUs) using the kmer searching method and the Silva bacterial database (www.arb-silva.de/). All OTUs had a taxonomic cut-off of ≥97%. Classification was performed using the Ribosomal Database Project (RDP) reference sequence files and the Wang method (Wang et al., 2007). The RDP MultiClassifier script was used for determination of OTUs (phylum to genus) and species level taxonomies were determined using USEARCH (Edgar, 2010). To account for potential bias introduced by differences in sequence depth, samples were rarefied to the smallest OTU read count (n = 1,750). OTUs with <10 reads across the dataset were considered rare taxa and were grouped (taxonomy_species X). Statistical modelling was performed using the top twenty species observed which accounted for >97% of the total reads. Diversity indices (e.g., non-parametric Shannon index, Inverse Simpson index and species observed (S_Obs_) were calculated using the Vegan package within R.

### Quantification of CVF Cytokine Concentrations and Leukocyte Counts

CVF was extracted from the soft cup as previously published by this group (Short et al., 2018). Multiplex chemiluminescent assays (V-plex Human Pro-inflammatory cytokine panel, Meso Scale Discovery^®^ (MSD)) were used to measure concentrations of ten cytokines: IFN-γ, IL-1β, IL-2, IL-4, IL-6, IL-8, IL-10, IL-12, IL-13, and TNF-α according to the manufacturer’s instructions on duplicate samples at a four-fold dilution in extraction buffer containing Protease-inhibitor cocktail, previously described (Short et al., 2018).

Light microscopy was performed on 21 samples for which a paired dry high vaginal smear was available to grade polymorphonuclear leucocyte count by ordinal scale: 0, 1–5, 6–10, 11–20, 21–30, and 31+ per high-powered field over an average of 3 fields.

### Statistical Analyses

Hierarchical clustering analysis (Ward linkage) was performed on rarefied species level count data using ClustViz (Metsalu and Vilo, 2015) to facilitate the classification of all samples into six vaginal microbiota types (VMB 1–6) on the basis of relative abundance profiles. For comparison by HIV status analyses were conducted on a cross section of second trimester samples from the 53 PWLWH (16.0–26.9 weeks) and 22 HUPW (22.0–22.1 weeks). The Statistical Analysis of Metagenomic Profiles (STAMP) software package was used to explore mean proportions of vaginal bacterial genera and species by HIV status on in the second trimester samples (Parks and Beiko, 2010). Statistical significance of differences was tested using the Welsh test and corrected using Benjamini-Hochberg False Discovery Rate.

#### PWLWH Cohort Analyses

Within PWLWH, statistical comparison of the proportions of term and preterm delivery in each VMB were made using Fisher’s exact test in the cross section of second trimester samples. For the 49 women for whom 79 matched CVF samples were available, the proportion with elevated cytokine concentrations [defined as the upper quartile of results (Arnold et al., 2016)] were compared by VMB group using Chi-Squared test in the statistical software package, SPSS (version 24; IBM, Armonk NY, USA). These analyses were performed for each of the three timepoints. Cytokine concentrations were log transformed to normalise their distribution and correlated with bacterial diversity indices and species abundance using Pearson’s correlation co-efficient in SPSS. Leucocyte count was correlated with log transformed cytokine concentration using Spearman’s correlation co-efficient in SPSS.

#### Longitudinal Data

For PWLWH women who had two or more samples, VMB stability was examined pictorially by plotting VMB type as a function of sample collection time point with the corresponding Inverse Simpson Index. The proportion of repeated samples where VMB transition was observed were compared by initial second trimester VMB classification using Chi-Squared test in SPSS. Median gestational age at delivery by occurrence of VMB transition were compared by the Mann Whitney U test

The relationship between bacterial species and gestational age at delivery was also explored using hierarchical multiple linear regression analysis in SPSS. The model was adjusted for maternal age, BMI and ethnicity, log transformed bacterial abundance was inputted as the second model factor, with patient ID included as a random effect. Fold increase of fit of the model of gestational age at delivery and individual bacterial species abundance is presented alongside the Beta Estimate value.

## Results

### Study Participant Characteristics

In this prospective study of pregnant women consenting to genital tract sampling, metataxonomics profiling was undertaken on vaginal swabs collected from 53 PWLWH and 22 HUPW. The median age of PWLWH (34 years, range 21–42) was similar to that of HUPW (32 years, range 26–39), p = 0·06 (**Table 1**). Compared to HUPW, PWLWH were more likely to be Black [81% (43/53) vs 23% (5/22)], and less likely to be Asian [4% (2/53) vs 41% (9/22)] or Caucasian [9% (5/53) vs 36% (8/22)] (all comparisons p <0·0001). PWLWH and HUPW were both non-smokers. PWLWH a mean BMI that was in the overweight category [28 (range 18–44)], no BMI data were available for HUPW.

**Table 1:** Clinical characteristics of participants by HIV status.

Characteristic	HIV-1 infected pregnant women, n = 53	Uninfected pregnant women, n = 22	p value
Maternal age,			0.06
Median (range)	34 (21–42)	32 (26–39)	
Ethnicity, n (%)			
Caucasian Black Asian Other	5 (9) 43 (81) 2 (4) 3 (6)	9 (41) 5(23) 8 (36) 0	<0.0001 <0.0001 <0.0001
BMI, kg/m^2^ (IQR) Missing, n	27 (22–31) 7	Na	–
Smoker, n (%)			
Yes No	1 (2) 52 (98)	0 22 (100)	0.71
Parity, n (%)			–
Nulliparous Multiparous	20 (38) 33 (62)	na	
PTB risk factors, n (%)			–
Yes No	14 (26) 39 (74)	0 22 (100)	
Prior PTB, n Cervical surgery Fibroids HTN Diabetes	13 (25) 4 (8) 2 (4) 1 (2) 2 (4)	0 na na 0 0	–
Median gestational age at delivery,	39 (38–40)	40 (39–41)	0.03
weeks (IQR) Missing, n	4		
Birth outcome, n (%)			
Term Preterm Other Missing, n	43 (89) 6 (12) 1 IUD 4	22 (100) 0 0 0	–

The mean CD4 count for PWLWH was within the normal range [668 cells/mcL (range 356–1,505)] and most women had a fully supressed infection with plasma HIV loads of <40 copies/mL at the time of sampling [79% (42/53)] (**Table 2**). Forty-one PWLWH had conceived on combination antiretroviral therapy (cART) with the non-nucleoside reverse transcriptase inhibitor, Efavirenz (EFZ) with the nucleotide analogue Tenofovir disoproxil fumarate (TDF) and the nucleoside analogue Emtricitabine (FTC) the most prescribed regimen (**Supplementary Table 1**). Twelve women initiated cART during pregnancy, with five initiating a Protease Inhibitor (PI) based and four Integrase-based regimen.

**Table 2:** Immune parameters and ART in HIV-1 infected pregnant women.

HIV infection specific clinical details	
CD4+ cell count at entry /mcL, median (IQR)	632 (505–770)
Percentage of total T cells expressing CD4+ at entry, median (IQR)	38 (32–43)
HIV viral load at entry copies/ml, median (range)	<40 (<20–16,113)
ART at conception, n (%)	
Yes No	41 (77) 12 (23)
ART class, n (%)	
PI based Non-PI based	20 (38) 33 (62)
NRTI backbone, n (%)	
FTC/TDF ABC/3TC	33 (62) 20 (38)

There were no preterm births to HUPW where as six (12%) PWLWH delivered preterm. Of these three delivered following the spontaneous onset of preterm labour at 31, 32, and 35 weeks gestational age (GA). Two were delivered by Caesarean section because of evidence of fetal compromise in the context of a small for gestational age baby. One was delivered by Caesarean section because of evidence of fetal compromise, although her baby was normally grown. There was also one stillbirth at 39 weeks.

### The Vaginal Microbiota During the 2^nd^ Trimester Differs Between PWLWH and HUPW

The mean number of sequences per sample for PWLWH was 28,692 (range 8,015–206,599) and for HUPW 14,974 (range 1,750–27,111). A total of 102 bacterial taxa were identified across all samples with *L. iners*, *L. crispatus* and *G. vaginalis* being the three most abundant species observed in the dataset. Ward clustering of bacterial species data enabled participant samples (n = 139 samples, HIV = 117, uninfected = 22) to be assigned to one of six vaginal microbiota groups (VMB); I (*L. crispatus* dominant), II (*L. gasseri* dominant), III (*L. iners* dominant), IIIb (*L. iners* with presence of *Gardnerella vaginalis)*, IV (Diverse, high proportions of *Atopobium, Gardnerella, Prevotella* spp. and others), and V (*L. jensensii* dominant) (**Supplementary Figure 1**).

**Figure 1:**
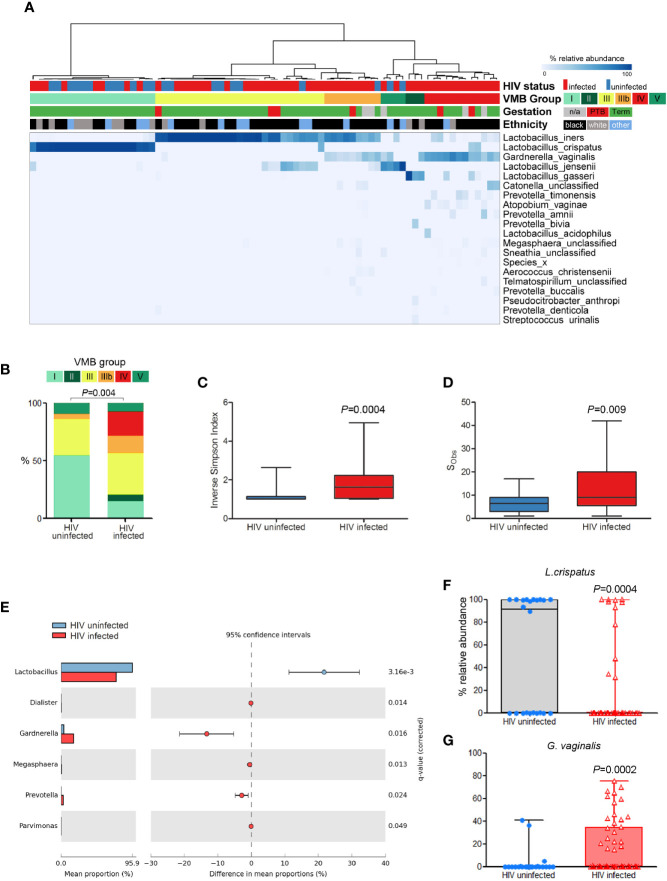
Comparison of vaginal microbiota composition of PWLWH (n = 53) and HUPW (n = 22) sampled during the second trimester of pregnancy. **(A)** Hierarchical clustering (Ward linkage) of relative abundance data of vaginal bacterial species identified six major vaginal microbiota (VMB) groups, with HIV status, ethnicity, and gestation at delivery for each patient presented above the heat map. **(B)** Proportions of VMB types differed significantly between PWLWH and HUPW with the former characterised by increased bacterial **(C)** diversity and **(D)** richness. **(E)** Compared to HUPW, samples from PWLWH had lower relative abundance of *Lactobacillus* spp. and *Gardnerella* spp. which were largely driven by significantly lower proportions of *L crispatus* **(F)** and higher proportions of *G. vaginalis* **(G)**.

Analysis of a cross-section (n = 75; PWLWH = 53, HUPW = 22) of samples collected in the second trimester of pregnancy (**Figure 1A**) showed that vaginal microbiota from HUPW at this stage of gestation were largely dominated by *Lactobacillus* spp. with 54% (12/22) having VMB I-type communities, 32% (7/22) VMB III, 9% (2/22) VMB V with only one VMB IIIb sample observed. In contrast, the predominant VMB in PWLWH was VMB III (36%, 19/53), followed by VMB IV (21%,11/53), VMB IIIb (15%, 8/53) and VMB I (15%, 8/53) (**Figure 1B**). A similar proportion of self-reported Black and Caucasian women were observed to have VMB I, however, VMB III and IV compositions were mainly observed in Black women. Consistent with these observations, PWLWH had significantly greater vaginal bacterial diversity (**Figure 1C**) and richness (**Figure 1D**) compared to HUPW. PWLWH had higher relative abundance of *Gardnerella* and *Prevotella* species compared to HUPW and a lower relative abundance of *Lactobacillus* spp., which remained significant after correction for multiple comparisons (**Figure 1E**). At species level, these differences were largely driven by higher relative abundance of *G. vaginalis* (p = 0·0002) and lower levels of *L. crispatus* (p = 0·0004) (**Figures 1F, G**).

## PWLWH Cohort

### High Bacterial Diversity and Mixed Anaerobes are Associated with Increased Local Inflammation in PWLWH

**Figure 2:**
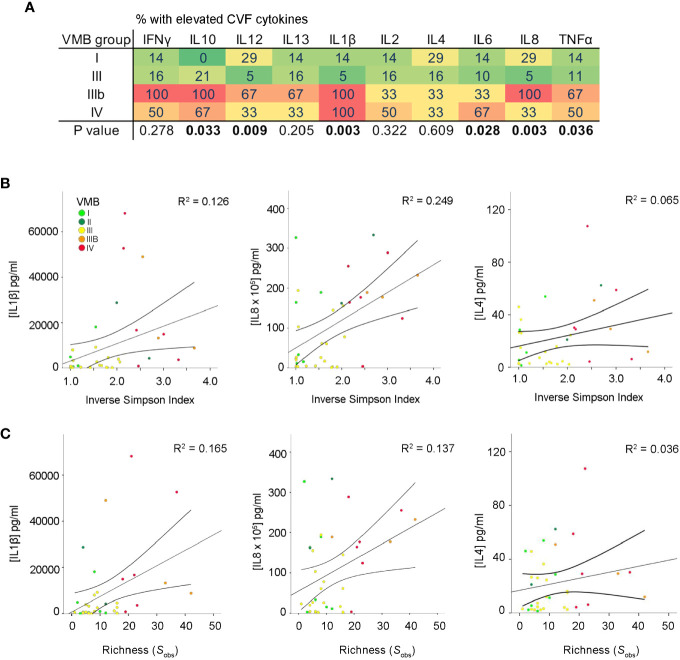
Increased vaginal microbial diversity and richness associates with local inflammation in PWLWH. **(A)** Heat map displaying proportion of t1 samples with elevated CVF cytokines in each VMB group defined by the cytokine concentration being in the upper quartile. A positive correlation between both IL-1β and IL-8 was observed between **(B)** α-diversity (Inverse Simpson Index) and **(C)** richness (species observed; S_obs_) in PWLWH. No such correlations were observed for IL-4 or the remaining cytokines assessed.

Cervicovaginal cytokine levels were determined in a total of 79 matched samples collected from 49 PWLWH (t1: n = 37, t2: n = 15, and t3: n = 27). Pro-inflammatory cytokine concentrations of IL-1β and TNF-α were significantly elevated in VMB IIIb and IV compared to other VMBs at the first second trimester timepoint (t1) (**Figure 2A**) with a similar trend seen at the third trimester timepoint (t3) but not at t2 (**Supplementary Figure 2**). Consistent with this, a positive correlation between both IL-1β and IL-8 levels and vaginal bacterial diversity and richness was observed in PWLWH (**Figures 2B, C**).

Mean proportions of major anaerobic species (*Aerococcus christensenii*, *Atopobium vaginalis*, *BVAB1*, *Dialister* spp.*, Gardnerella* spp., *Prevotella* spp. and *Sneathia* spp.) were positively correlated with CVF levels of pro-inflammatory cytokines IFN-γ, IL-1β, IL-8, and TNF-α [**Table 3** (t1, n = 37), **Supplementary Table 2** (t1–3, n = 79)]. IL-1β was also positively correlated with mean proportions of *L. gasseri* and *L. jensenii* at t1, which was maintained for *L. jensenii* in the longitudinal samples. In a subset of matched samples where high power field microscopy was available (n = 21), total leucocyte count was found to significantly correlate with pro-inflammatory IL-1β (ρ = 0.506, p = 0·023) and a trend towards association with IFN-γ (ρ = 0.423, p = 0·063) was observed.

**Table 3:** Association between vaginal microbiota and selected cervicovaginal pro-inflammatory cytokines during the second trimester of HIV-infected women.

	IL-1β		IL-8		IFN-γ		TNF-α	
	r	p	r	p	r	p	r	p
*L. iners*	−0.361	0.03	−0.406	0.013	−0.024	0.890	−0.210	0.213
*L. crispatus*	−0.165	0.328	−0.054	0.749	−0.102	0.547	−0.109	0.520
*L. jensenii*	0.583	< 0.0001	0.295	0.076	0.064	0.708	0.312	0.060
*L. gasseri*	0.350	0.034	0.329	0.047	0.434	0.007	0.307	0.065
BVAB1	0.080	0.639	0.108	0.526	−0.498	0.020	0.042	0.805
*G. vaginalis*	0.585	< 0.0001	0.296	0.075	0.066	0.697	0.313	0.059
*Prevotella* spp.	0.227	0.176	0.222	0.188	0.176	0.296	0.139	0.413
*Atopobium vaginalis*	0.406	0.013	0.261	0.919	−0.020	0.909	0.288	0.084
*Megasphaera* spp.	0.041	0.811	−0.032	0.853	−0.222	0.186	−0.040	0.813
*L. acidophilus*	0.184	0.276	0.145	0.392	−0.187	0.267	0.162	0.337
*Snaethia* spp.	0.211	0.209	0.202	0.229	0.085	0.619	0.070	0.681
*Aerococcus christensenii*	0.344	0.037	0.248	0.139	−0.120	0.478	0.265	0.115
*Telmatospirillum unclass*	−0.023	0.893	−0.064	0.704	−0.018	0.916	−0.190	0.260
*Anaerococcus* spp.	0.158	0.351	0.184	0.275	0.091	0.594	−0.028	0.872
*Dialister* spp.	0.370	0.04	0.370	0.024	0.188	0.264	0.340	0.039

Correlation analysis was performed using relative abundance data of vaginal bacterial species and matched log transformed CVF cytokine levels collected from HIV-infected women. r = Pearson’s correlation co-efficient.

### VMB I Was the Most Stable and VMB IIIb Was the Least Stable During Pregnancy

**Figure 3:**
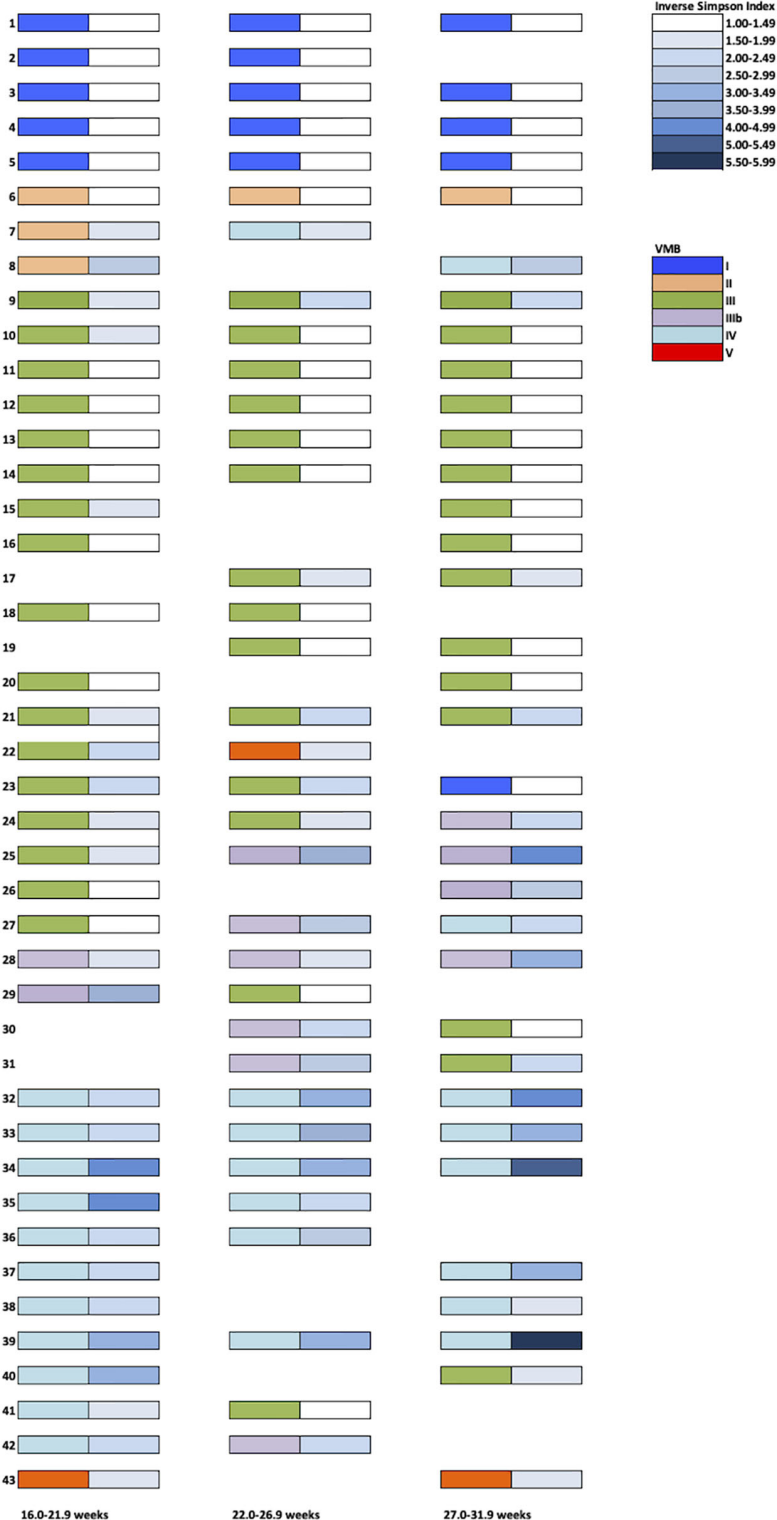
Vaginal Microbiota Group (VMB) profiles throughout pregnancy in a UK PWLWH cohort. Each sample was assigned a VMB indicated by the left-sided coloured rectangles. Corresponding Inverse Simpson Indices are presented on the right-side (white- low diversity and dark blue- high diversity).

Forty-three PWLWH had two or more consecutive vaginal samples (**Figure 3**). Women whose VMB were classified as I or V on their first second trimester sample, remained in the same microbiota group throughout the sampling period. PWLWH whose VMB was group III in the second trimester remained stable (13/19) or transitioned (6/19 (32%)) to IIIb (4) or other lactobacillus predominant VMBs I (1) or V (1). VMB IIIb during the second trimester was the least stable (4/6 (67%)) with transitions to III (3) and IV (1). Similarly (2/3 (67%) of VMB II transitioned to IV (2). Whilst there were three cases (3/11 (27%)) where VMB IV reverted from high diversity to lower diversity VMB III (2) and IIIb (1), more cases (8/11) remained in this high diversity microbiota type, many of which demonstrated an increase in α diversity (Inverse simpson index) through pregnancy. The observation that the proportion of PWLWH whose VMBs remained stable across repeated sampling differed according to their initial second trimester VMB grouping, approached but did not reach statistical significance, p = 0.09. The median gestational age at delivery for PWLWH in whom VMB transition occurred was slightly lower [38.2 weeks (IQR 37.1–40.1)] than in women whose VMB remained stable [39.0 weeks (IQR 38.2–40.4], this did not reach statistical significance p = 0.11.

### Associations Between Vaginal Microbiota and Gestational Age at Delivery in PWLWH

**Figure 4:**
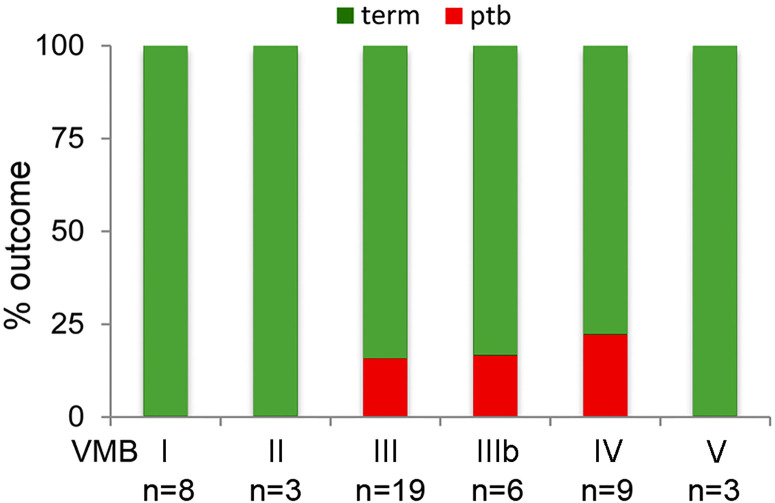
Delivery outcomes by VMB group in PWLWH. In those PWLWH where delivery outcome were available (n = 46/53), 16% (3/19) of women in VMB III delivered preterm, 17% (1/6) of women in VMB IIIb and 22% (2/9) of women in VMB IV. No preterm births were recorded in those women with VMB I, II, and V.

Examination of birth outcomes in PWLWH showed that all PTB in the cohort (n = 6) occurred in women with VMB III, IIIb, and IV vaginal microbiota compositions (**Figure 4**). All women with VMB I, II and V type profiles delivered at term. Hierarchical linear regression was used to model the relationship between relative abundance of vaginal bacterial species with gestational age at delivery, following adjustment for ethnicity, maternal age, BMI, with patient ID inputted as a random effect. *Prevotella* spp.*, Sneathia* spp. and *Dialister* spp. were all found to inversely associate with gestational age at delivery, whereas *L. crispatus* had a positive association with gestational age at delivery (**Table 4**). A trend towards an inverse association between gestational age at delivery at *L.gasseri* and *L.jensenii* was also observed but did not reach significance level.

**Table 4:** Hierarchical linear regression modelling of vaginal bacterial species and gestational age at delivery.

Species	Fold Change	Estimate	Std.Error	F value	Q value
***Lactobacillus iners***	2.78	−0.27	0.16	−1.67	0.09
***Lactobacillus crispatus***	4.38	0.34	0.16	2.09	0.04
***Lactobacillus jensenii***	3.09	−0.29	0.16	−1.76	0.08
***Lactobacillus gasseri***	2.85	−0.43	0.26	−1.69	0.09
***BVAB1***	1.79	−0.85	0.63	−1.34	0.24
***Gardnerella vaginalis***	3.25	−0.29	0.16	−1.8	0.07
***Prevotella* spp.**	5.55	−0.52	0.22	−2.36	0.02
***Atopobium vaginae***	0.05	−0.1	0.46	−0.21	0.83
***Megasphaera* spp.**	0.02	−0.51	0.86	−0.59	0.56
***Lactobacillus acidophilus***	13.73	3.04	0.82	3.71	0.17
***Sneathia* spp.**	7.45	−1.24	0.45	−2.73	0.01
***Aerococcus christensenii***	1.85	−0.72	0.53	−1.36	0.18
***Telmatospirillum unclass.***	0.9	−1.77	1.86	−0.95	0.41
***Anearococcus* spp.**	1.48	−0.83	0.68	−1.22	0.26
***Dialister* spp.**	7.62	−1.27	0.46	−2.76	0.01

Beta estimate value is presented alongside fold increase of fit of the model of gestational age at delivery with individual bacterial species abundance inputted as a secondary factor. Following adjustment for ethnicity, maternal age, BMI, with patient ID inputted as a random effect, Prevotella spp., Sneathia spp., and Dialister spp. were all found to inversely associate with gestational age at delivery, whereas L. crispatus had a positive association with gestational age at delivery.

## Discussion

PWLWH are at increased risk of poor pregnancy outcomes such as PTB through a poorly defined pathogenesis. Substantial evidence now implicates vaginal microbiota composition during pregnancy with subsequent risk of PTB (Beigi et al., 2007; Brown et al., 2018; Brown et al., 2019; Elovitz et al., 2019; Fettweis et al., 2019). In this study, we report that vaginal microbiota of PWLWH was characterised by increased prevalence of high diversity community compositions compared to uninfected women delivering at term. These communities were typically enriched for *G. vaginalis* and deplete in *Lactobacillus* species, particularly *L. crispatus*, which has been reported to provide protection against adverse pregnancy outcomes including PTB in a number of patient cohorts (Kindinger et al., 2017; Brown et al., 2018; Fettweis et al., 2019). In addition to our study of European-based PWLWH, increased vaginal bacterial diversity has been recently observed in PWLWH in Zimbabwe and Zambia (Price et al., 2019; Gudza-Mugabe et al., 2020), suggesting it is characteristic of HIV infection in both non-pregnant and pregnant women.

Longitudinal study of VMB composition in this cohort of PWLWH women revealed stability in the small proportion with VMB I but equally demonstrated high degrees of instability especially in VMB II, IIIB and IV between the second and third trimester. These data are consistent with earlier work in both pregnant and non-pregnant women that has shown the *L. crispatus* dominant communities are the most stable and *L. iners* and *L. gasseri* dominant compositions are more likely to transition to higher diversity communities, that associate with PTB (Verstraelen et al., 2009; MacIntyre et al., 2015; Kindinger et al., 2017).

High-diversity vaginal microbiota and/or colonisation by pathogens often associated with bacterial vaginosis have been widely reported to increase risk of PTB in uninfected pregnant women (Beigi et al., 2007; Brown et al., 2018; Brown et al., 2019; Elovitz et al., 2019; Fettweis et al., 2019). Such findings are consistent with the model of ascending vaginal pathogen colonisation and associated inflammation (infiltration of innate immune cells and expression of high concentrations of pro-inflammatory cytokines) in gestational tissues as a mechanistic driver of premature labour and birth (Goldenberg et al., 2008). Here we show in PWLWH that elevated cervicovaginal pro-inflammatory cytokines are associated with increased vaginal bacterial diversity and richness as well as the relative abundance of specific BV-associated pathobionts including *G. vaginalis, Atopobium, Dialister, Prevotella*, and *Sneathia* species and the number of polymorphonuclear leucocytes, most likely neutrophils. The association observed between IL-1β and *L.gasseri* and *L.jensenii* was unexpected especially as these species are typically considered part of a healthy vaginal microbiota. The significance of this finding is uncertain and could be the result of type 1 error as the number of PWLWH with these VMB was small.

Notably, all PTB in this cohort of PWLWH, regardless of being spontaneous or induced secondary to obstetric indications, occurred in women with high diversity or BV-type VMBs (III, IIIb, and IV). Exploring gestational age at delivery as a continuum, higher abundance of anaerobic bacteria *Prevotella* spp., *Sneathia* spp., and *Dialister* spp. were associated with earlier delivery whereas *L. crispatus* was associated with later delivery.

These findings differ to a recent study of PWLWH by Gudza-Mugabe and colleagues who concluded they did not observe a relationship between vaginal microbiota, vaginal cytokine levels, and PTB preterm birth (Gudza-Mugabe et al., 2020). This difference may be partly explained by differences in study design, particularly the gestational age of sampling and method of collection of genital tract fluid. While we focused on investigating samples collected in the second trimester [median 21 weeks (IQR 20–22)], the median sampling of patients in the Gudza-Mugabe and co-workers study was 29 weeks [IQR 25–33]. It is possible that any relationship between vaginal microbiota and local inflammatory status and gestational length is lost at these later gestational timepoints, however we were able to demonstrate similar trends in a smaller number of third trimester samples. In addition, our sampling method for cytokine measurement, the menstrual cup, collects a greater representative sample of cellular and cytokine expression in the lower female genital tract than a single vaginal swab. In spite of these differences, Gudza-Mugabe and colleagues were able to identify significant associations (R > 0.3) between six bacterial taxa and vaginal cytokine concentrations that withstood correction for multiple testing. Also in accordance with our findings, a recent study by Lopez and co-workers reported increased plasma concentrations of soluble CD14 and liposaccharide-binding-protein (markers of bacterial translocation) in the first trimester in PWLWH who went on to experience PTB compared to PWLWH delivering at term and uninfected pregnancies (Lopez et al., 2016).

The analysis of the vaginal microbiota at species level and integration of matched cytokine profiles in early gestation is a strength of our exploratory study. However, a relatively small number of PTB cases in PWLWH limited our ability to draw strong conclusions between microbiota composition, local inflammation and PTB risk in this cohort. We were not sufficiently powered to look at the effects of antiretroviral on PTB, an initial objective, but a strength of our study was the inclusion of a CD4 entry cut off of ≥350 cells/ml to limit any bias from underlying immune suppression. Differences in gestation age at delivery by individual drugs or drug classes could not be examined due to the diversity of therapies used. Most PWLWH conceived on cART limiting exploration of the potential role of initiation of therapy in PTB.

The PWLWH cohort was enriched for women self-reporting Black race compared to uninfected controls thus making it harder to attribute HIV status alone as the cause for differences in vaginal microbiota composition. Black race has previously been associated with increased colonisation of *L. iners* and high-diversity compositions during pregnancy, however recent data from the Human Microbiome Project suggests that women of African ancestry experience a rapid shift towards *Lactobacillus* spp. dominance early in pregnancy, which associates with simplification of the metabolic capacity of the microbiome (Serrano et al., 2019). Moreover, the linear regression model we used to identify a relationship between BV-associated pathobionts and earlier gestational age at delivery was corrected for ethnicity, maternal age and BMI.

Whilst spontaneous and iatrogenic labour are clearly distinguishable, the link observed here between dysbiosis and PTB regardless of its iatrogenic or spontaneous nature suggests that common pathways may underlie both. Fetal growth restriction is associated with some of the same biomarkers as spontaneous PTB (Kirkegaard et al., 2010). There is a well-known association between spontaneous PTB and growth restriction *in utero*; in general preterm babies tend to be small for gestational age (Lackman et al., 2001). Vaginal dysbiosis is also a risk factor for miscarriage (Al-Memar et al., 2020), which suggests a potential effect upon decidual function and placentation. It is possible that the adverse effect of having a specific bacterial profile may act very early in pregnancy. It is notable that of the four PTB in our cohort that did not follow spontaneous labour, three had clear evidence of abnormal fetal growth and fetal compromise and the fourth had fetal heart rate abnormalities despite normal fetal growth, each of which suggests significantly compromised placental function.

### Generalisability and Conclusion

We conclude that high rates of unfavourable VMB are associated with cervico-vaginal inflammation in pregnancy which in turn contribute to the high rate of PTB experienced by PWLWH despite numerical restoration of CD4 T-cells.

Our women were recruited in the UK, however the high percentage of self-reported Black race, many from Sub-Saharan Africa, potentially widens the relevance of our results. Further elucidation of the infectious triggers to PTB in PWLWH, the interaction of antiretroviral therapy including the effects of new therapies on the microbiota, in addition to focusing of obstetric management of high risk women e.g. risk stratification tools, are imperative to reduce its global impact.

## Data Availability Statement

The RNASeq data are available in the SRA: PRJEB41429. The study name is ena-STUDY-CUMICRO-18-11-2020-15:38:11:823-609.

## Ethics Statement

The studies involving human participants were reviewed and approved by NHS Health Research Authority National Research Ethics Service (NRES) Committee approval REC 13/LO/0107 (HIV infected pregnant women) and REC 14/LO/0328 (uninfected pregnant women). The patients/participants provided their written informed consent to participate in this study.

## Author Contributions

C-ES, PB, GT, and DM conceived and designed the study. Patient recruitment and sample collection were undertaken by C-ES, RB, and RQ. Experiments and data collection were performed by C-ES, RB, RQ, and YL. Data processing, analyses, and interpretation were performed by C-ES, AS, PB, GT, and DM. All figures and tables were generated by C-ES and DM. C-ES wrote the first draft of the manuscript and all authors contributed critical revisions to the paper, interpretation of the results and approved the final version. All authors contributed to the article and approved the submitted version.

## Funding

This study was funded by the Wellcome Trust Clinical PhD Programme (C-ES, grant no. WT/102757/Z/13/Z, the Medical Research Council (DM, grant no. MR/L009226/1), the March of Dimes European Preterm Birth Research Centre at Imperial College London and the National Institute of Health Research (NIHR) Imperial Biomedical Research Centre (BRC) (PB and DM, grant no. P45272). The views expressed are those of the authors and not necessarily those of the NHS, the NIHR, or the Department of Health.

## Conflict of Interest

PB reports personal fees and shares and stock ownership in ObsEva Pharmaceuticals, personal fees from GlaxoSmithKline that are both outside the submitted work. PB and DM have a patent for microRNA markers to predict cervical shortening and preterm birth issued again outside of the submitted work.

The remaining authors declare that the research was conducted in the absence of any commercial or financial relationships that could be construed as a potential conflict of interest.

## References

[B1] Al-Memar M. Bobdiwala S. Fourie H. Mannino R. Lee Y. S. Smith A. (2020). The association between vaginal bacterial composition and miscarriage: a nested case-control study. BJOG an Int. J. Obstetr. Gynaecol. 127 (2), 264–274. 10.1111/1471-0528.15972 PMC6972675 31573753

[B2] Arnold K. B. Burgener A. Birse K. Romas L. Dunphy L. J. Shahabi K. (2016). Increased levels of inflammatory cytokines in the female reproductive tract are associated with altered expression of proteases, mucosal barrier proteins, and an influx of HIV-susceptible target cells. Mucosal Immunol. 9 (1), 194–205. 10.1038/mi.2015.51 26104913

[B3] Beigi R. H. Yudin M. H. Cosentino L. Meyn L. A. Hillier S. L. (2007). Cytokines, pregnancy, and bacterial vaginosis: comparison of levels of cervical cytokines in pregnant and nonpregnant women with bacterial vaginosis. J. Infect. Diseases 196 (9), 1355–1360. 10.1086/521628 17922400

[B4] Blencowe H. Cousens S. Oestergaard M. Z. Chou D. Moller A. B. Narwal R. (2012). National, regional, and worldwide estimates of preterm birth rates in the year 2010 with time trends since 1990 for selected countries: a systematic analysis and implications. Lancet 379 (9832), 2162–2172. 10.1016/S0140-6736(12)60820-4 22682464

[B5] Borgdorff H. Tsivtsivadze E. Verhelst R. Marzorati M. Jurriaans S. Ndayisaba G. F. (2014). Lactobacillus-dominated cervicovaginal microbiota associated with reduced HIV/STI prevalence and genital HIV viral load in African women. ISME J. 8 (9), 1781–1793. 10.1038/ismej.2014.26 24599071 PMC4139719

[B6] Brown R. G. Marchesi J. R. Lee Y. S. Smith A. Lehne B. Kindinger L. M. (2018). Vaginal dysbiosis increases risk of preterm fetal membrane rupture, neonatal sepsis and is exacerbated by erythromycin. BMC Med. 16 (1), 9. 10.1186/s12916-017-0999-x 29361936 PMC5782380

[B7] Brown R. G. Al-Memar M. Marchesi J. R. Lee Y. S. Smith A. Chan D. (2019). Establishment of vaginal microbiota composition in early pregnancy and its association with subsequent preterm prelabor rupture of the fetal membranes. Transl. Res. 207, 30–43. 10.1016/j.trsl.2018.12.005 30633889 PMC6489901

[B8] Edgar R. C. (2010). Search and clustering orders of magnitude faster than BLAST. Bioinformatics 26 (19), 2460–2461. 10.1093/bioinformatics/btq461 20709691

[B9] Elovitz M. A. Gajer P. Riis V. Brown A. G. Humphrys M. S. Holm J. B. (2019). Cervicovaginal microbiota and local immune response modulate the risk of spontaneous preterm delivery. Nat. Commun. 10 (1), 1305. 10.1038/s41467-019-09285-9 30899005 PMC6428888

[B10] Fettweis J. M. Serrano M. G. Brooks J. P. Edwards D. J. Girerd P. H. Parikh H. I. (2019). The vaginal microbiome and preterm birth. Nat. Med. 25 (6), 1012–1021. 10.1038/s41591-019-0450-2 31142849 PMC6750801

[B11] Fowler M. G. Qin M. Fiscus S. A. Currier J. S. Flynn P. M. Chipato T. (2016). Benefits and Risks of Antiretroviral Therapy for Perinatal HIV Prevention. New Engl. J. Med. 375 (18), 1726–1737. 10.1056/NEJMoa1511691 27806243 PMC5214343

[B12] Frank J. A. Reich C. I. Sharma S. Weisbaum J. S. Wilson B. A. Olsen G. J. (2008). Critical evaluation of two primers commonly used for amplification of bacterial 16S rRNA genes. Appl. Environ. Microbiol. 74 (8), 2461–2470. 10.1128/AEM.02272-07 18296538 PMC2293150

[B13] Gilleece D. Y. Tariq D. S. Bamford D. A. Bhagani D. S. Byrne D. L. Clarke D. E. (2019). British HIV Association guidelines for the management of HIV in pregnancy and postpartum 2018. HIV Med. 20 (Suppl 3), s2–s85. 10.1111/hiv.12720 30869192

[B14] Goldenberg R. L. Culhane J. F. Iams J. D. Romero R. (2008). Epidemiology and causes of preterm birth. Lancet 371 (9606), 75–84. 10.1016/S0140-6736(08)60074-4 18177778 PMC7134569

[B15] Grosch-Woerner I. Puch K. Maier R. F. Niehues T. Notheis G. Patel D. (2008). Increased rate of prematurity associated with antenatal antiretroviral therapy in a German/Austrian cohort of HIV-1-infected women. HIV Med. 9 (1), 6–13. 10.1111/j.1468-1293.2008.00520.x 18199167

[B16] Gudza-Mugabe M. Havyarimana E. Jaumdally S. Garson K. L. Lennard K. Tarupiwa A. (2020). Human Immunodeficiency Virus Infection Is Associated With Preterm Delivery Independent of Vaginal Microbiota in Pregnant African Women. J. Infect. Dis. 221 (7), 1194–1203. 10.1093/infdis/jiz584 31722395 PMC7075414

[B17] Hay P. E. Lamont R. F. Taylor-Robinson D. Morgan D. J. Ison C. Pearson J. (1994). Abnormal bacterial colonisation of the genital tract and subsequent preterm delivery and late miscarriage. Bmj 308 (6924), 295–298. 10.1136/bmj.308.6924.295 8124116 PMC2539287

[B18] Hummelen R. Fernandes A. D. Macklaim J. M. Dickson R. J. Changalucha J. Gloor G. B. (2010). Deep sequencing of the vaginal microbiota of women with HIV. PloS One 5 (8), e12078. 10.1371/journal.pone.0012078 20711427 PMC2920804

[B19] Kindinger L. M. MacIntyre D. A. Lee Y. S. Marchesi J. R. Smith A. McDonald J. A. (2016). Relationship between vaginal microbial dysbiosis, inflammation, and pregnancy outcomes in cervical cerclage. Sci. Trans. Med. 8 (350), 350ra102. 10.1126/scitranslmed.aag1026 27488896

[B20] Kindinger L. M. Bennett P. R. Lee Y. S. Marchesi J. R. Smith A. Cacciatore S. (2017). The interaction between vaginal microbiota, cervical length, and vaginal progesterone treatment for preterm birth risk. Microbiome 5 (1), 6. 10.1186/s40168-016-0223-9 28103952 PMC5244550

[B21] Kirkegaard I. Uldbjerg N. Petersen O. B. Torring N. Henriksen T. B. (2010). PAPP-A, free beta-hCG, and early fetal growth identify two pathways leading to preterm delivery. Prenatal Diagnosis 30 (10), 956–963. 10.1002/pd.2593 20721873

[B22] Klatt N. R. Cheu R. Birse K. Zevin A. S. Perner M. Noel-Romas L. (2017). Vaginal bacteria modify HIV tenofovir microbicide efficacy in African women. Science 356 (6341), 938–945. 10.1126/science.aai9383 28572388

[B23] Lackman F. Capewell V. Richardson B. daSilva O. Gagnon R. (2001). The risks of spontaneous preterm delivery and perinatal mortality in relation to size at birth according to fetal versus neonatal growth standards. Am. J. Obstetr. Gynecol. 184 (5), 946–953. 10.1067/mob.2001.111719 11303203

[B24] Liu L. Hill K. Oza S. Hogan D. Chu Y. Cousens S. (2016). “Levels and Causes of Mortality under Age Five Years,” in Black R. E. Laxminarayan R. Temmerman M. Walker N., editors. Reproductive, Maternal, Newborn, and Child Health. Disease Control Priorities, third edition, volume 2 Washington, DC: World Bank. 10.1596/978-1-4648-0348-2 27227205

[B25] Lopez M. Figueras F. Coll O. Gonce A. Hernandez S. Lonca M. (2016). Inflammatory Markers Related to Microbial Translocation Among HIV-Infected Pregnant Women: A Risk Factor of Preterm Delivery. J. Infect. Dis. 213 (3), 343–350. 10.1093/infdis/jiv416 26265778

[B26] MacIntyre D. A. Chandiramani M. Lee Y. S. Kindinger L. Smith A. Angelopoulos N. (2015). The vaginal microbiome during pregnancy and the postpartum period in a European population. Sci. Rep. 5, 8988. 10.1038/srep08988 25758319 PMC4355684

[B27] Metsalu T. Vilo J. (2015). ClustVis: a web tool for visualizing clustering of multivariate data using Principal Component Analysis and heatmap. Nucleic Acids Res. 43 (W1), W566–W570. 10.1093/nar/gkv468 25969447 PMC4489295

[B28] Parks D. H. Beiko R. G. (2010). Identifying biologically relevant differences between metagenomic communities. Bioinformatics 26 (6), 715–721. 10.1093/bioinformatics/btq041 20130030

[B29] Pepin J. Deslandes S. Giroux G. Sobela F. Khonde N. Diakite S. (2011). The complex vaginal flora of West African women with bacterial vaginosis. PloS One 6 (9), e25082. 10.1371/journal.pone.0025082 21949860 PMC3176826

[B30] Powis K. M. Kitch D. Ogwu A. Hughes M. D. Lockman S. Leidner J. (2011). Increased risk of preterm delivery among HIV-infected women randomized to protease versus nucleoside reverse transcriptase inhibitor-based HAART during pregnancy. J. Infect. Dis. 204 (4), 506–514. 10.1093/infdis/jir307 21791651 PMC3144169

[B31] Price J. T. Vwalika B. Hobbs M. Nelson J. A. E. Stringer E. M. Zou F. (2019). Highly diverse anaerobe-predominant vaginal microbiota among HIV-infected pregnant women in Zambia. PloS One 14 (10), e0223128. 10.1371/journal.pone.0223128 31577818 PMC6774526

[B32] Reimers L. L. Mehta S. D. Massad L. S. Burk R. D. Xie X. Ravel J. (2016). The Cervicovaginal Microbiota and Its Associations With Human Papillomavirus Detection in HIV-Infected and HIV-Uninfected Women. J. Infect. Dis. 214 (9), 1361–1369. 10.1093/infdis/jiw374 27521363 PMC5079369

[B33] Serrano M. G. Parikh H. I. Brooks J. P. Edwards D. J. Arodz T. J. Edupuganti L. (2019). Racioethnic diversity in the dynamics of the vaginal microbiome during pregnancy. Nat. Med. 25 (6), 1001–1011. 10.1038/s41591-019-0465-8 31142850 PMC6746180

[B34] Short C. E. Taylor G. P. (2014). Antiretroviral therapy and preterm birth in HIV-infected women Vol. 12 (Expert review of anti-infective therapy). Taylor and Francis 293–306. 10.1586/14787210.2014.885837 24502750

[B35] Short C. S. Quinlan R. Bennett P. Shattock R. J. Taylor G. P. (2018). Optimising the collection of female genital tract fluid for cytokine analysis in pregnant women. J. Immunol. Methods 458, 15–20. 10.1016/j.jim.2018.03.014 29625077 PMC5981004

[B36] Spear G. T. Sikaroodi M. Zariffard M. R. Landay A. L. French A. L. Gillevet P. M. (2008). Comparison of the diversity of the vaginal microbiota in HIV-infected and HIV-uninfected women with or without bacterial vaginosis. J. Infect. Diseases 198 (8), 1131–1140. 10.1086/591942 18717638 PMC2800037

[B37] Thorne C. Patel D. Newell M. L. (2004). Increased risk of adverse pregnancy outcomes in HIV-infected women treated with highly active antiretroviral therapy in Europe. Aids 18 (17), 2337–2339. 10.1097/00002030-200411190-00019 15577551

[B38] UNAIDS (2018). UNAIDS DATA 2018. Available at: http://www.unaids.org/sites/default/files/media_asset/unaids-data-2018_en.pdf.

[B39] Uthman O. A. Nachega J. B. Anderson J. Kanters S. Mills E. J. Renaud F. (2017). Timing of initiation of antiretroviral therapy and adverse pregnancy outcomes: a systematic review and meta-analysis. Lancet HIV 4 (1), e21–e30. 10.1016/S2352-3018(16)30195-3 27864000

[B40] Verstraelen H. Verhelst R. Claeys G. De Backer E. Temmerman M. Vaneechoutte M. (2009). Longitudinal analysis of the vaginal microflora in pregnancy suggests that L. crispatus promotes the stability of the normal vaginal microflora and that L. gasseri and/or L. iners are more conducive to the occurrence of abnormal vaginal microflora. BMC Microbiol. 9, 116. 10.1186/1471-2180-9-116 19490622 PMC2698831

[B41] Wang Q. Garrity G. M. Tiedje J. M. Cole J. R. (2007). Naive Bayesian classifier for rapid assignment of rRNA sequences into the new bacterial taxonomy. Appl. Environ. Microbiol. 73 (16), 5261–5267. 10.1128/AEM.00062-07 17586664 PMC1950982

