# Supplementary material for: Lactobacillus-Depleted Vaginal Microbiota in Pregnant Women Living With HIV-1 Infection Are Associated With Increased Local Inflammation and Preterm Birth
Source: Front Cell Infect Microbiol. 2021 Feb 11;10:596917. doi: 10.3389/fcimb.2020.596917 (PMC7905210; doi:10.3389/fcimb.2020.596917)
Supplement: Supplementary file 1 [file DataSheet_1.docx]

Supplementary Material

**Supplementary Figure 1**


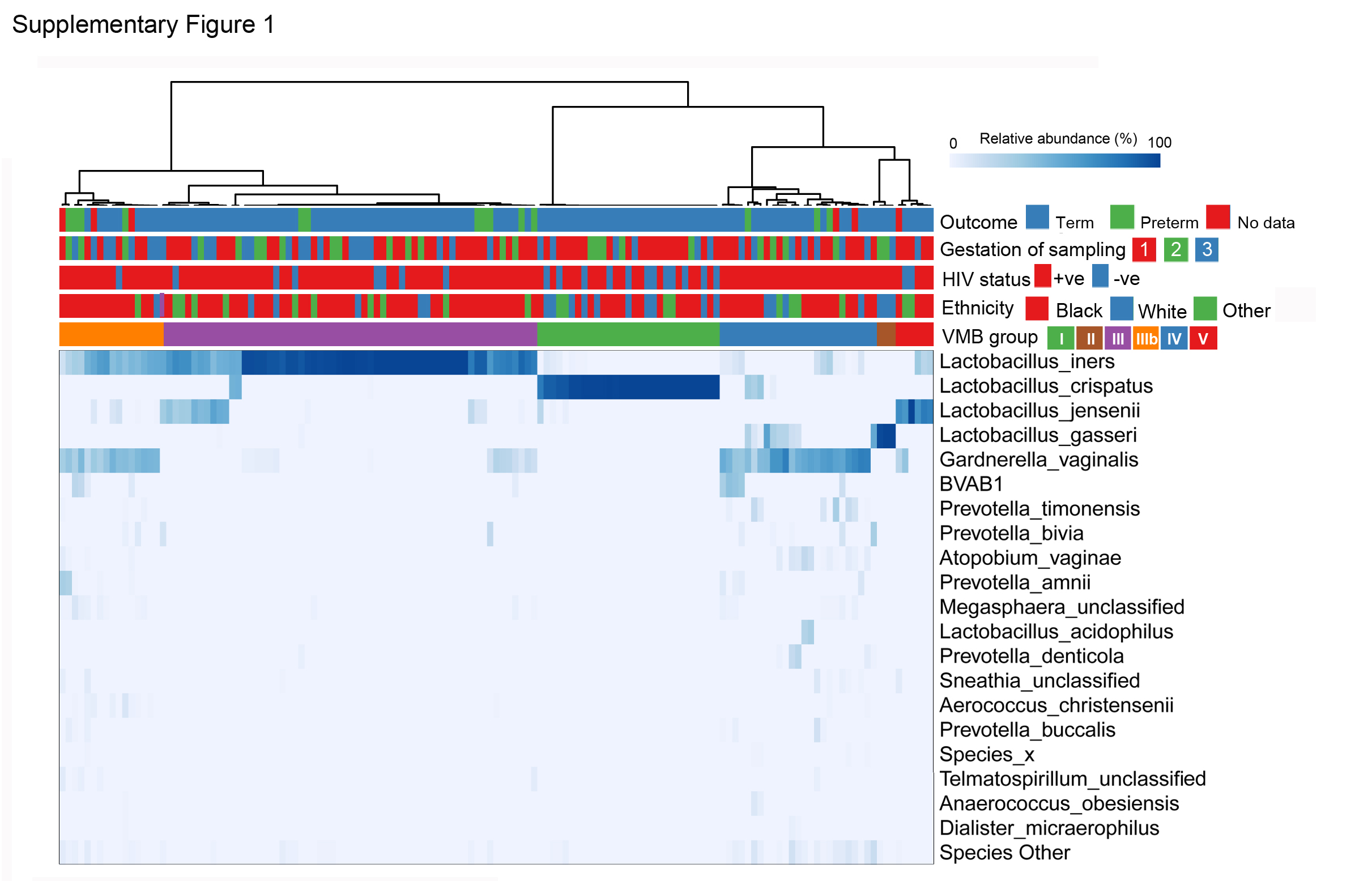


**Hierarchical clustering (Ward linkage) of relative abundance data of vaginal bacterial species in all sample from the study cohort (n=139).** Six major vaginal microbiota (VMB) groups were identified, with HIV status, ethnicity and gestation at delivery for each patient presented above the heat map.

**Supplementary Table 1: ART exposure in HIV-1 infected pregnant women**

| Study group n=53 | Conceiving on  PI-based n=15 | Conceiving on  Non-PI n=26 | Initiating  PI n=5 | Initiating  Non-PI n=7 |
| --- | --- | --- | --- | --- |
| Base drug | Boosted Atazanavir: 10  Boosted Darunavir: 5 | Efavirenz: 14  Nevirapine: 5  Rilpiverine: 2  Raltegravir: 5 | Boosted Atazanavir: 4  Boosted Darunavir: 1 | Trizivir: 3  Raltegravir: 2  Dolutegravir: 2 |

Trizivir – Abacavir, zidovudine, lamivudine

**
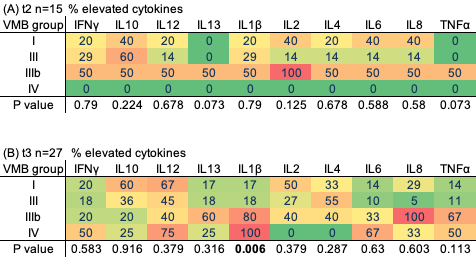
 Supplementary Figure 2: Heat maps displaying proportion of matched CVF samples with elevated cytokines in each VMB group**

(A) t2 samples (22.0-21.9 weeks) (B) t3 samples (27.0-31.9 weeks), elevated cytokine concentration defined as being in the upper quartile

**Supplementary table 2 Associations between vaginal microbiota and log transformed cervicovaginal pro-inflammatory cytokines in pregnancy**

r = pearson’s correlation co-efficient. longitudinal data (n=79 matched microbiome & cvf cytokine, from 49 women, weeks 16-31), * p<0.0001

|  | **IL-1β** | | **IL-8** | | **IFN-γ** | | **TNF-α** | |
| --- | --- | --- | --- | --- | --- | --- | --- | --- |
|  | **r** | **p** | **r** | **p** | **r** | **p** | **r** | **p** |
| ***L.* iners** | **-0.255** | **0.024** | **-0.312** | **0.005** | -0.030 | 0.794 | -0.195 | 0.085 |
| ***L. crispatus*** | -0.234 | 0.038 | -0.008 | 0.947 | -0.090 | 0.429 | -0.132 | 0.947 |
| ***L.* jensenii** | **0.567** | ***** | **0.313** | **0.005** | 0.083 | 0.465 | **0.364** | **0.001** |
| ***L.* gasseri** | 0.127 | 0.228 | 0.175 | 0.124 | 0.141 | 0.214 | 0.205 | 0.070 |
| ***BVAB1*** | 0.182 | 0.109 | 0.110 | 0.335 | -0.191 | 0.091 | 0.105 | 0.357 |
| ***G.vaginalis*** | **0.566** | ***** | **0.313** | **0.005** | 0.086 | 0.454 | **0.362** | **0.001** |
| ***Prevotella spp.*** | **0.431** | ***** | **0.319** | **0.004** | **0.313** | **0.005** | **0.317** | **0.004** |
| ***Atopobium vaginalis*** | **0.391** | ***** | **0.241** | **0.032** | 0.119 | 0.297 | **0.282** | **0.012** |
| ***Megasphaera spp.*** | 0.078 | 0.494 | -0.026 | 0.818 | **-0.264** | **0.019** | -0.050 | 0.661 |
| ***L. acidophilus*** | 0.105 | 0.356 | 0.095 | 0.403 | -0.133 | 0.243 | 0.101 | 0.376 |
| ***Snaethia spp.*** | **0.246** | **0.029** | 0.184 | 0.105 | 0.027 | 0.814 | 0.107 | 0.350 |
| ***Aerococcus christensenii*** | **0.345** | **0.002** | **0.262** | **0.020** | 0.037 | 0.746 | **0.254** | **0.024** |
| ***Telmatospirillum_unclass*** | -0.038 | 0.737 | -0.080 | 0.481 | -0.073 | 0.525 | -0.194 | 0.086 |
| ***Anaerococcus spp.*** | **0.257** | **0.022** | **0.228** | **0.043** | 0.026 | 0.821 | **0.164** | **0.150** |
| ***Dialister spp.*** | **0.476** | ***** | **0.357** | ***** | **0.372** | **0.001** | **0.393** | ***** |

**
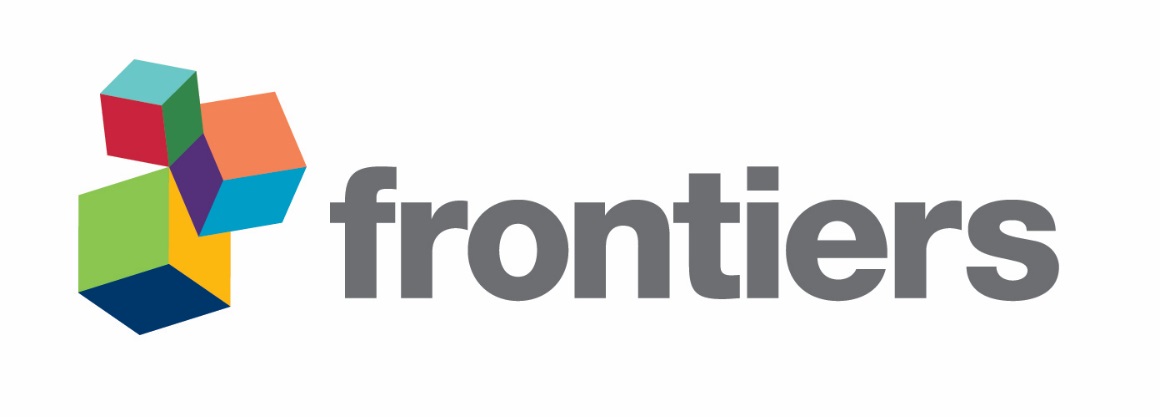
**
